# An Atypical Presentation of a Severe Case of Anaplasma Phagocytophilum

**DOI:** 10.7759/cureus.23224

**Published:** 2022-03-16

**Authors:** Sameer Kandhi, Haider Ghazanfar, Zaheer A Qureshi, Harika Kalangi, Abhilasha Jyala, Esther S Arguello Perez

**Affiliations:** 1 Internal Medicine, BronxCare Health System, Bronx, USA; 2 Internal Medicine, Icahn School of Medicine at Mount Sinai, New York, USA; 3 Infectious Disease, BronxCare Health System, Bronx, USA

**Keywords:** anaplasmosis, black-legged ticks, infectious disease, ticks, elevated liver functions, thrombocytopenia, leucopenia, anaplasma

## Abstract

We report a case of a 79-year-old male presenting to a South Bronx hospital with complaints of fever, shortness of breath, severe thrombocytopenia, hematuria, elevated liver enzymes, and acute renal failure. The patient rapidly progressed to acute hypoxic respiratory failure requiring mechanical ventilation. Treatment was delayed for six days because the tick-borne disease was not considered in the differential. Empirical treatment of tick-borne illnesses should be considered in the proper clinical setting, and travel history should be relevant in any patient presenting with fever. Delay in appropriate treatment results in the onset of more severe illness.

## Introduction

Anaplasmosis, also known as human granulocytic anaplasmosis (formerly known as human granulocytic ehrlichiosis), is a tick-borne illness caused by the anaplasma phagocytophilum [1]. In the last two decades, we have seen a tremendous increase in the number of anaplasmosis cases reported to CDC, from 348 cases in 2000 to a peak of 5,762 cases in 2017 [2,3]. The elderly population, immunocompromised patients, patients with advanced human immunodeficiency virus (HIV) infection, and organ transplant recipients are at risk of severe outcomes from the disease. Delay in appropriate treatment results in the onset of more severe illness involving respiratory failure, bleeding diathesis, multiorgan failure, and death [4]. In the following, we describe a severe case of anaplasmosis whose diagnosis was delayed.

## Case presentation

A 79-year-old male with medical comorbidities of hypertension, benign prostate hypertrophy status post transurethral prostatectomy, cerebrovascular accident, and pulmonary hypertension presented to the Emergency Department (ED) at our hospital in New York City, complaining of fever for three days, shortness of breath, generalized weakness, constipation, and hematuria. He had been in his usual state of health five days prior when he began experiencing constipation and generalized body weakness after eating in a restaurant with his friends. After two days, he began to experience shortness of breath with a fever of 101 Fahrenheit along with progressive constipation and poor oral intake to the point where he was tolerating only fluids. On the presentation day, the wife found blood in the patient's urine while at home and called the Emergency Medical Services (EMS).

In the ED, the patient was found with a heart rate of 103 beats per minute, a temperature of 98.2 F, blood pressure of 139/92 mm of Hg, respiratory rate of 30 breaths per minute, and oxygen saturation of 92% on room air. His lung auscultation revealed bilateral rales, and his cardiovascular examination showed a systolic murmur in the second intercostal space. His abdomen was distended, soft, and non-tender. He had 2+ pitting edema in the bilateral lower extremities. The patient was alert and oriented to time, place, and person. His initial laboratory values are shown in Table 1.

**Table 1:** Initial laboratory test results

Lab Tests	Day 1 of Hospitalization	Day 6	At Time of Discharge	Reference Range
White Blood Cell count	10.7 k/µL	15.9 k/µL	10.4 k/µL	4.8-10.8 k/µL
Neutrophil Count	9.3 k/µL	7.9 k/µL	8.8 k/µL	1.5-8.0 k/µL
Lymphocyte Count	0.6 k/µL	5.7 k/µL	1.0 k/µL	1.0-4.8 k/µL
Red Blood Cell Count	4.10 MIL/µL	2.72 MIL/µL	3.01 MIL/µL	4.50-5.90 MIL/µL
Hemoglobin	13.0 g/dL	7.9 mg/dL	9.6 mg/dL	12.0-16.0 g/dL
Hematocrit	37.2%	25.3%	29%	42-51%
Platelet	24 k/µL	90 k/µL	196 k/µL	150-400 k/µL
General Chemistry				
Sodium, Serum	134 mEq/L	151 mEq/L	147 mEq/L	135-145 mEq/L
Potassium, Serum	3.4 mEq/L	4.2 mEq/L	3.6 mEq/L	3.5-5.0 mEq/L
Blood Urea Nitrogen, Serum	33 mg/dL	155 mg/mL	30 mg/mL	8-26 mg/dL
Creatinine, Serum	2.0 mg/dL	4.3 mg/dL	0.6 mg/dL	0.5-1.5 mg/dL
Hepatic Function Panel				
Bilirubin, Serum total	3.3 mg/dL	0.9 mg/dL	0.2 mg/dL	0.2-1.1 mg/dL
Bilirubin, Serum Direct Conjugated	2.5 mg/dL	0.7 mg/dL	< 0.2 mg/dL	0.0-0.3 mg/dL
Alkaline Phosphatase, Serum	64 unit/L	33 units/L	108 units/L	56-155 unit/L
Aspartate Transaminase, Serum	163 unit/L	218 units/L	11 units/L	9-48 unit/L
Alanine Aminotransferase, Serum	64 unit/L	98 units /L	9 units /L	5-40 unit/L
Lactic acid Level	4.0 mmoles/L	3.1 mmoles/L	0.8 mmoles/L	0.5-1.6 mmoles/L
Fibrinogen	358 mg/dL	590 mg/dL		185-450 mg/dL
D-dimer Assay, Plasma	3,244 ng/mL	5,869 ng/mL		0-230 ng/mL
Lactate Dehydrogenase, Serum	>1,000 unit/L			110-210 unit/L
Haptoglobin, Serum	<10.0 mg/dL			30-200 mg/dL
Ferritin	>5,000 ng/mL			13-150 ng/mL
Urine Toxicology	Negative			

**Figure 1:**
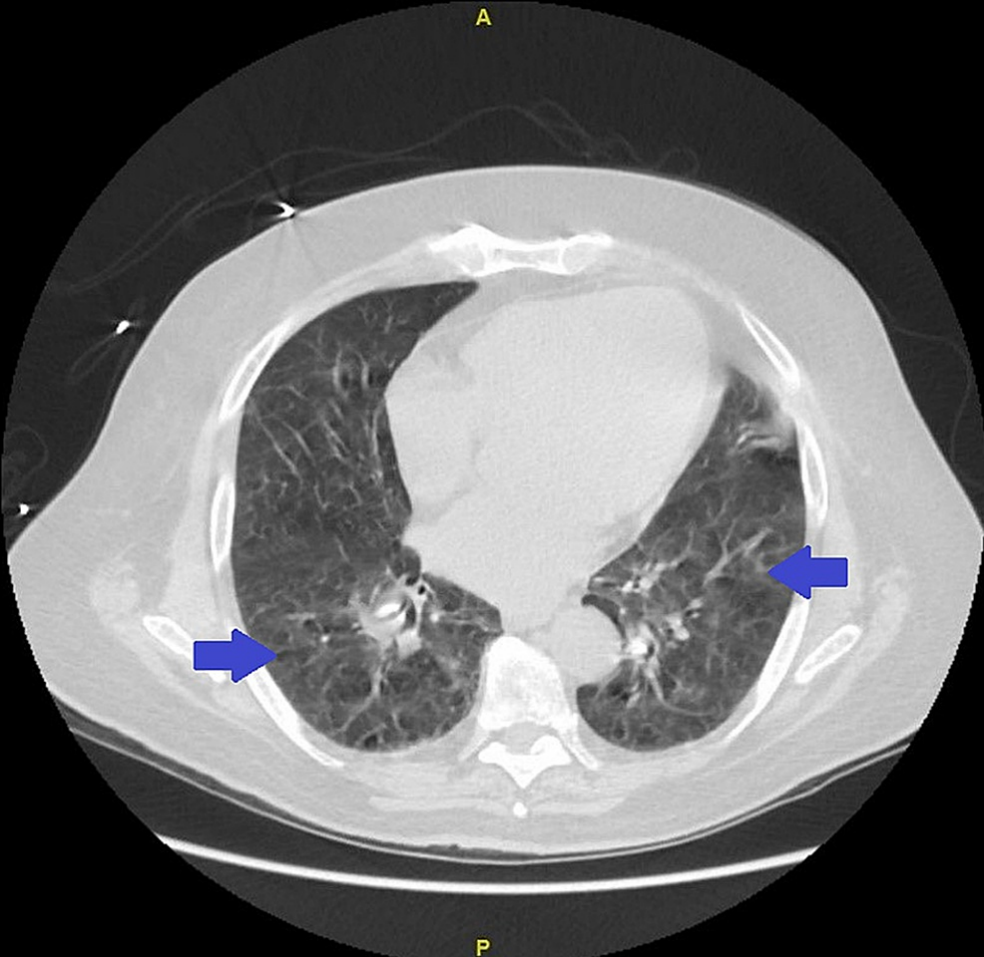
Computed tomography (CT) of chest showing increased lung markings with scattered bilateral ground-glass opacities (GGO) (blue arrow)

**Figure 2:**
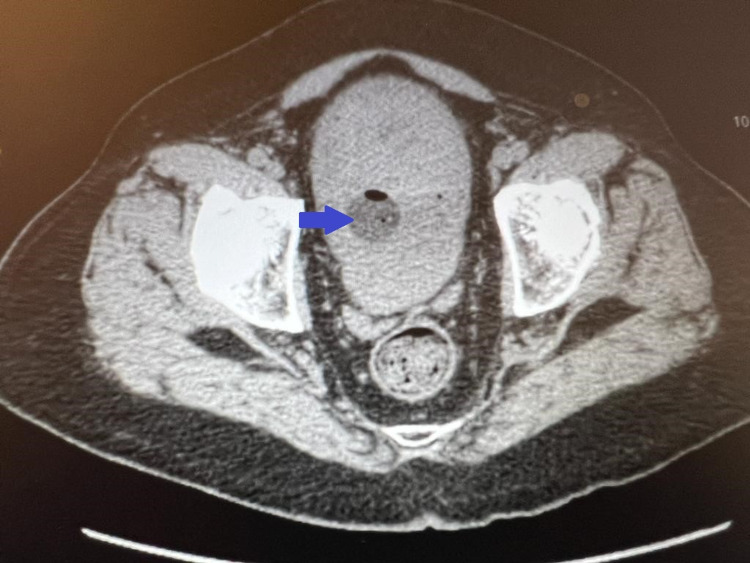
Computed tomography (CT) of abdomen and pelvis showing a large filling defect within the urinary bladder suspicious for a blood clot (blue arrow)

On admission, the chest's computed tomography (CT) showed increased lung markings with scattered ground-glass opacities. This has been presented in Figure 1. The abdomen and pelvis CT showed a large filling defect within the urinary bladder, suggestive of a possible large blood clot and fluid collections adjacent to the anterior dome of the bladder, suggestive of a probable bladder diverticulum. This has been presented in Figure 2. The Urology service was consulted, and the clinician recommended inserting a Foley catheter for gross hematuria and bladder irrigation to remove the clots.

The patient developed acute hypoxic respiratory failure on admission and was intubated. He was started on intravenous (IV) ceftriaxone and azithromycin for suspected community-acquired pneumonia (CAP). On day 2, post-intubation, ceftriaxone was switched to IV vancomycin and piperacillin/tazobactam for broader coverage. He was found to be persistently febrile despite being on broad-spectrum antibiotics. On day 6 of hospitalization, infectious disease was consulted and recommended upgrade to meropenem and to add doxycycline, because, when asked about possible exposure to a tick bite, the patient's wife stated that the patient regularly walked in a park in New Jersey, and had had a recent tick bite.

The patient's hospital course was further complicated by new-onset systolic heart failure with a reduced ejection fraction of 27% atrial flutter with supraventricular tachycardia requiring amiodarone. The patient started to have severe worsening anemia, requiring multiple packed red blood cell (PRBC) transfusions secondary to overt upper gastrointestinal bleeding. The patient was evaluated by gastroenterology service for the same and was offered esophagogastroduodenoscopy (EGD), which the patient's wife declined. He received a total of 9 PRBC transfusions during the entire hospital course.

The patient's laboratory investigation revealed negative blood, urine, respiratory cultures. Peripheral smear was negative for schistocytes and had normal ADAMS-13 activity. He was found to have a Tick-borne molecular panel positive for anaplasmosis polymerase chain reaction (PCR) along with positive serology for acute Lyme's disease. Leptospira was ruled out, and malaria and babesia smears were negative. The patient completed a 14-day course of doxycycline. The patient improved after the treatment with fever defervescence occurring on day 2 of treatment with doxycycline. His acute kidney injury resolved, and platelet count and liver enzymes returned to baseline. However, due to prolonged respiratory failure, the patient had to undergo tracheostomy and percutaneous endoscopic gastrostomy (PEG). The patient was discharged to the nursing home after an extended course of hospitalization in stable condition.

## Discussion

Early symptoms of anaplasmosis are mild and usually occur within 1-5 days. These include fever, chills, headache, muscle pain, malaise, nausea, abdominal pain, cough, and confusion [5,6]. Patients usually develop symptoms five to 14 days after a tick bite [7], and up to 75% of patients will report exposure to ticks [3]. Lack of pathognomonic rash in many instances compared with other tick-borne infections also contributes to the delayed diagnosis of the disease. The rash is only seen in approximately 6% to 10% of cases or with those co-infected with Borrelia burgdorferi [7,8].

In our case, the patient did not have a rash and initially reported nonspecific symptoms of subjective fever, malaise, and constipation. The exposure to ticks was revealed only later in the clinical course after direct questioning the caregiver about tick exposure. Our patient had presented to the hospital on day 5 of his symptoms. Moreover, the primary team did not consider his diagnosis of suspected tick-borne illness and initiation of doxycycline. The primary team was anchored in the diagnosis of pneumonia and stayed locked in the initial diagnosis without considering further differential. Another contributing factor could be the primary team's lack of knowledge about tick-borne diseases rarely encountered in a city hospital.

Furthermore, the team did not take travel history initially to suspect that the patient was at risk of a tick-borne disease. This failure to take the travel history may have resulted from a bias toward the patient. Given the patient's advanced age, with a history of stroke, and residing in an urban area, the team might not think the patient could be taking walks in a park where ticks are present. Laboratory data suggestive of leukopenia, thrombocytopenia, and transaminase elevations are essential clues to the diagnosis [4,7,8]. Our patient presented with fever with a temperature of 101F, severe thrombocytopenia (24 k/µL), elevated liver enzymes (AST of 163 µ/L), acute kidney injury (creatinine 2.0 mg/dL), which -- except for the leucopenia -- were all suggestive of anaplasmosis.

Severity sufficient for hospitalization is observed in 36% to 56% of symptomatic patients [3,6]. It is associated with older age, higher neutrophil counts, lower lymphocyte counts, anemia, the presence of morulae in leukocytes, or underlying immune suppression [6,9]. Our patient had a higher neutrophil count, lower lymphocyte count, and anemia. Approximately 7% to 17% of patients require intensive care [3,8]. The fatality rate has been reported to be 1.2% among those 20 to 39 years of age [10], in which delayed diagnosis and treatment were risk factors [8]. Three percent may develop life-threatening complications [3] requiring hospitalization and ICU admission, including acute respiratory distress syndrome (ARDS), acute renal failure, coagulopathy, rhabdomyolysis, myocarditis, and hemorrhage. Our patient had acute respiratory distress syndrome, acute renal failure, hemorrhage, coagulopathy, and heart failure. The delay in the initiation of treatment for anaplasmosis and his advanced age might have contributed in his case to all these complications.

Ixodes scapularis is a well-known tick vector for babesia microti, anaplasma, and borrelia species. These pathogens' co-infection rates have been reported from 2% to 13% [3,11]. A study on 311 patients showed that patients with co-infection had more symptoms than patients with anaplasmosis alone [5]. Our patient had positive serologies for acute Lyme and positive PCR for anaplasmosis suggestive of co-infection, which can be considered a probable reason for severe presentation. Cases of anaplasmosis can occur during any part of the year. However, a significant proportion of them are reported during the summer months of June and July, typically corresponding to the season of nymphal black-legged ticks, with a smaller peak usually in October to November when adult black-legged ticks are most likely active [12]. Our patient presented during July, which corresponds to the known peak season of incidence for tick-borne illness. Traditionally, in patients presenting with unexplained fevers or tick bites, the illness is more frequently suspected in patients who live closer to tick habitats like woods river valleys. We argue that patients who present with unexplained fevers to urban hospitals during summer and early fall should be tested and empirically treated for suspected tick-borne infections in the exemplary clinical scenario.

Diagnosing anaplasmosis through staining blood smears from peripheral blood, bone marrow, or CSF to detect morulae is a well-known rapid diagnostic feature among practicing clinicians but is seen in only about 20% of infected patients [3]. No characteristic morulae were detected on the peripheral blood smear of our patient on admission, which further contributed to the delay in the diagnosis. Doxycycline remains the drug of choice and first-line treatment for anaplasmosis in adults and pediatric cases. 100 mg twice daily for 10 to 14 days is recommended by the CDC to cover the possibility of concurrent Lyme disease infection. Using antibiotics other than doxycycline increases the risk of severe illness and patient death. Observational reports indicate that delay in treatment is associated with a poor outcome and increased length of hospital stay [10,13]. Our patient has been treated with doxycycline 100 mg twice daily for 14 days. Unfortunately, the delay in diagnosis and treatment, along with his advanced age and severe presentation on admission, contributed to his poor outcome and increased length of hospital stay.

## Conclusions

Patients who present with fever, thrombocytopenia, leukopenia, and elevated liver enzymes, especially between spring and early fall, even to a city hospital, should be considered for the possibility of a tick-borne disease. It is essential always to obtain a detailed clinical history that can reveal epidemiological risk factors for tick-borne illness in the exemplary clinical scenario. Delayed treatment can cause catastrophic complications.
